# Evaluation of the relationships between inflammatory markers and processing speed in recent-onset and chronic schizophrenia: a polygenic risk score study

**DOI:** 10.1192/j.eurpsy.2026.11269

**Published:** 2026-07-31

**Authors:** M. V. Alfimova, V. V. Plakunova, V. Golimbet

**Affiliations:** Clinical genetics laboratory, Mental Health Research Center, Moscow, Russian Federation

## Abstract

**Introduction:**

Empirical evidence suggests a link between elevated circulating levels of inflammatory markers and cognitive deficits in schizophrenia (Sz). Specifically, negative correlations have been found between interleikin-6 (IL-6) and C-reactive protein (CRP) levels and processing speed. However, further studies are needed to explore the complex interplay between immune mechanisms and cognition during the Sz course. Given that peripheral immunomarker levels are state-dependent, studies using genetic proxies of immune markers may add valuable information on the relationships between inflammation and cognitive functions.

**Objectives:**

The study aimed to evaluate the relation between polygenic predictors of circulating CRP and IL-6 levels and processing speed in early and chronic stages of Sz.

**Methods:**

269 patients with Sz spectrum disorders (ICD-10, F20-25; 54% women; age range 16-66 years), who underwent whole-genome genotyping and cognitive testing, were included. The patients were divided into two groups: with recent-onset Sz (RoSz: illness duration ≤ 5 years, n=135) and with chronic Sz (CrSz: illness duration > 5 years, n=124). Semantic verbal fluency (VF) was used as a measure of processing speed. Polygenic risk scores (PRS) for CRP, Il-6 and IL-6RA levels as well as for several potential confounders were calculated using the Michigan Imputation Server. Hierarchical linear regression was used to assess an incremental contribution of the set of inflammatory markers’ PRS to explaining VF variance compared to the null model. The latter included sex, PANSS negative syndrome scores, and PRS for Sz (Sz-PRS), Alzheimer disease (AD-PRS) and fluid intelligence (FI-PRS), as well as two ancestry-related principal components.

**Results:**

The immune-related PRSs did not significantly improve the VF prediction in either group (RoSz: p=0.38, ΔR^2^=0.02; CrSz: p=0.47, ΔR^2^=0.02). Among the confounders, sex correlated with VF in both groups, with women scoring higher than men. Remarkedly, SZ-PRS was related to VF in the RoSz group (β=-1.53, SE=0.75, bootstrapping-based bias corrected accelerated (bca) p=0.04), while AD-PRS (β=-1.83, SE=0.89, bca p=0.03) and negative symptoms (β=-0.52, SE=0.14, bca p<0.001) were significant predictors of VF in the CrSz group.

**Conclusions:**

No significant association was found between PRS of CRP and IL-6 levels with verbal fluency in either recent-onset or chronic Sz. One explanation for the negative result may be that inflammation has a minor effect on the variability of cognitive deficits compared to other factors acting at different stages of the disease.

**Disclosure of Interest:**

None Declared

